# Hydration and nutrition care practices in stroke: findings from the UK and Australia

**DOI:** 10.1186/s12912-023-01575-4

**Published:** 2023-10-27

**Authors:** Colette Miller, Stephanie P. Jones, Munirah Bangee, Cintia Mayel Martinez-Garduno, Marian C. Brady, Dominique A. Cadilhac, Simeon Dale, Elizabeth McInnes, Sandy Middleton, Caroline L. Watkins, C. Elizabeth Lightbody

**Affiliations:** 1 Stroke Research Team, School of Nursing & Midwifery, University of Central Lancashire, Preston, PR1 2HE UK; 2 Nursing Research Institute - St Vincent’s Health Network Sydney, St Vincent’s Hospital Melbourne & Australian Catholic University, Sydney, Australia; 3 Nursing, Midwifery and Allied Health Professions Research Unit, Glasgow Caledonian University, Glasgow, UK; 4 Stroke and Ageing Research, Department of Medicine, School of Clinical Sciences at Monash Health, Monash University, VIC, Melbourne, Australia; 5 School of Nursing, Midwifery and Paramedicine, Australian Catholic University, VIC, Melbourne, Australia; 6 Lancashire Teaching Hospitals NHS Foundation Trust, Preston, UK

**Keywords:** Nursing, Hydration, Nutrition, Protocol, Survey, Stroke, United Kingdom, Australia, Cross-sectional study

## Abstract

**Background:**

Dehydration and malnutrition are common in hospitalised patients following stroke leading to poor outcomes including increased mortality. Little is known about hydration and nutrition care practices in hospital to avoid dehydration or malnutrition, and how these practices vary in different countries. This study sought to capture how the hydration and nutrition needs of patients’ post-stroke are assessed and managed in the United Kingdom (UK) and Australia (AUS).

**Aim:**

To examine and compare current in-hospital hydration and nutrition care practice for patients with stroke in the UK and Australia.

**Methods:**

A cross-sectional survey was conducted between April and November 2019. Questionnaires were mailed to stroke specialist nurses in UK and Australian hospitals providing post-stroke inpatient acute care or rehabilitation. Non-respondents were contacted up to five times.

**Results:**

We received 150/174 (86%) completed surveys from hospitals in the UK, and 120/162 (74%) in Australia. Of the 270 responding hospitals, 96% reported undertaking assessment of hydration status during an admission, with nurses most likely to complete assessments (85%). The most common methods of admission assessment were visual assessment of the patient (UK 62%; AUS 58%), weight (UK 52%; AUS 52%), and body mass index (UK 47%; AUS 42%). Almost all (99%) sites reported that nutrition status was assessed at some point during admission, and these were mainly completed by nurses (91%). Use of standardised nutrition screening tools were more common in the UK (91%) than Australia (60%). Similar proportions of hydration management decisions were made by physicians (UK 84%; AUS 83%), and nutrition management decisions by dietitians (UK 98%; AUS 97%).

**Conclusion:**

Despite broadly similar hydration and nutrition care practices after stroke in the UK and Australia, some variability was identified. Although nutrition assessment was more often informed by structured screening tools, the routine assessment of hydration was generally not. Nurses were responsible for assessment and monitoring, while dietitians and physicians undertook decision-making regarding management. Hydration care could be improved through the development of standardised assessment tools. This study highlights the need for increased implementation and use of evidence-based protocols in stroke hydration and nutrition care to improve patient outcomes.

**Supplementary Information:**

The online version contains supplementary material available at 10.1186/s12912-023-01575-4.

## Background

Dehydration and malnutrition are common in hospitalised patients and constitute a significant economic burden for health care providers, contributing to longer hospital stays and poorer outcomes [1, 2]. Over half of patients with stroke become dehydrated during their hospital stay, and their risk of malnutrition increases during the first ten days of admission [3, 4]. The wide range of physical, psychological, and social difficulties experienced post-stroke can affect patients’ ability to eat and drink independently. Factors that can affect eating and drinking include an inability to maintain head control; loss of upper limb motor control; difficulties chewing and swallowing; communication problems; and visual, perceptual, and attention deficits [5]. Consequently, many patients require assistance from others to eat and drink, further increasing their risk of inadequate fluid and food intake, and the development of associated complications [6]. Dehydration and malnutrition have been associated with an increased risk of death and dependency after stroke [7, 8].

Good hydration and nutrition are essential for recovery and rehabilitation following hospitalisation [9]. Unfortunately, despite the known consequences, post-stroke hydration and nutrition care have been neglected [10–12]. Recent research suggests dehydration and malnutrition are under-recognised and undertreated, with prevalence after stroke remaining high (dehydration (29–70%); malnutrition 28.7%) [13, 14]. While there is evidence that initial assessments of nutrition status are usually completed, follow-up actions such as offering nutritional supplements or repeated screening are less likely to be undertaken [15]. Approximately 20% of patients with stroke require alternative methods of feeding, but delays in the initiation of feeding, frequent dislodgement of nasogastric tubes, and other complications such as vomiting, reflux, and gastrointestinal bleeding, can contribute to declines in nutritional status [16, 17]. Similarly, the monitoring and assessment of hydration status are often undertaken intermittently in response to other clinical issues, and the timely provision of treatment can be affected by a lack of staff to assist with oral hydration, or to prescribe and monitor intravenous fluids [15, 18].

The importance of the assessment and management of hydration and nutrition are highlighted in national clinical guidelines, with UK clinical guidelines recommending that patients with acute stroke should be screened for risk of malnutrition on admission and have their hydration assessed within four hours of arrival [19]. The guidelines further recommend that hydration and nutrition status should be closely monitored [19]. These recommendations are echoed in national clinical guidelines from Australia and New Zealand [20, 21] which state that staff should be trained in the use of a structured nutrition screening tool, and refer to a dietitian where necessary. The advice regarding the assessment of hydration status is less clear with “multiple methods” of assessment being advocated to maintain “normal” hydration [19]. One explanation for this lack of clarity in guidelines may be the equivocal evidence base for the prevention and treatment of dehydration after stroke [13].

Evidence regarding the implementation of guideline recommendations in this area of stroke care is sparse. Audit data from an observational study in the UK has provided evidence that dehydration is common (56%), but assessment and diagnosis of dehydration are not routinely documented [22]. Authors of one qualitative study have explored the perceptions of healthcare professionals providing hydration care [18], reporting that staff felt insufficiently trained and required evidence-based protocols to deliver effective hydration care. There is no research specifically examining the patient experience of hydration and nutrition care. However, authors of a study exploring stroke survivors’ experiences of fundamental aspects of care, including eating and drinking, found patients often have distressing recollections of their experiences which are of detriment to their psychosocial and emotional wellbeing [23]. Therefore, further research is needed to better understand hydration and nutrition care practice post-stroke to provide direction on meeting the fundamental needs of patients in hospital after stroke.

The aim of this study was to examine and compare current hydration and nutrition care practice post-stroke in the UK and Australia.

## Methods

### Design

A cross-sectional survey to explore hydration and nutrition care practices following stroke in the UK and Australia, undertaken as part of a broader survey exploring oral health care practice, the findings of which have been reported previously [24] (The survey can be found in Supplementary File 1). The results of the survey are reported using the STrengthening the Reporting of OBservational studies in Epidemiology (STROBE) checklist [25] (Supplementary File 2).

#### Hospital selection

All hospitals known to provide stroke services (including stroke rehabilitation) in the UK and Australia were identified. Those in the UK were identified via the Royal College of Physicians’ Sentinel Stroke National Audit Programme (SSNAP) (England, Wales, and Northern Ireland) and via the Scottish Stroke Care Audit (Scotland). Hospitals in Australia were identified from the Stroke Foundation Organisational Survey [26] and the Stroke Foundation’s National Stroke Audit - Rehabilitation Services Report 2016 [27].

### Data collection

The survey was carried out in accordance with research governance regulations in each country and data were collected from April to November 2019. Survey methodology was employed as the most effective and efficient approach to generate insights regarding hydration and nutrition care practices of a large and geographically widespread target population [28]. The questionnaire was developed specifically for this study and was informed by expert knowledge, existing literature, and stroke guidelines [19–21]. Expert knowledge regarding the hydration and nutrition section of the survey was sought from an interdisciplinary clinical-academic writing group in the UK, funded by the British and Irish Association of Stroke Physicians, developing programmatic research in this area. In addition, the findings of preliminary exploratory research [18, 22], conducted by the writing group concurrently to the development of this survey but not yet published, informed the survey design. An expert panel of stroke clinicians (four from the UK and four from Australia) reviewed the questions, and response options for clarity, and to determine completion time. Data were collected to explore and describe: (1) demographics of the respondent, (2) characteristics of the stroke service, (3) hydration and nutrition practices (Sect. 7). Of the twelve questions exploring hydration and nutrition care practice, ten were closed/multiple-choice, but a free text option was available enabling respondents to provide an alternative answer to the choices given. Multiple-choice design was selected as such questions are less time-consuming for respondents, particularly important when considering the target population of senior healthcare professionals with hectic schedules and competing demands, and the pre-determined answers were derived from the existing evidence and guidelines to compare evidence with practice. For the remaining questions, respondents completed a five-point Likert scale (highly likely, likely, unsure, unlikely, highly unlikely). The five-point Likert scale was chosen as it is easily understood, allows for a wider range of responses, avoids forcing responses, and provides acceptable levels of reliability [29].

A key participant, the stroke unit coordinator/manager, stroke specialist nurse, or clinical lead, was identified within each participating stroke service, and their participation was voluntary. The key contact was encouraged to complete the survey in collaboration with other appropriate members of the team. Key contacts were sent an advance e-mail the day before the questionnaire was distributed as a pre-alert to the upcoming survey. Questionnaires were posted with a reply-paid envelope, and completed surveys could be returned in a pre-addressed stamped envelope or scanned and returned via fax or by email. Non-respondents were contacted up to five times (by email three times and by telephone twice) to optimise response rates as key participants were senior clinicians with competing priorities. Participants could withdraw from the study at any time and, should a participant decline to participate after receipt of an email or telephone call, no further attempts at follow-up were made.

### Data analysis

Data were entered in REDCap electronic data capture tools [30, 31] and prepared for statistical analysis using SPSS (IBM SPSS Statistics 26, IBM Corporation, Armonk, NY, USA). Categorical data were reported as proportions. Where there was non-response to specific questions within a returned questionnaire, the denominator has been adjusted accordingly. For the purpose of data analysis and reporting, Likert scale categories ‘Highly Likely’ and ‘Likely’ were combined to report a positive response, and ‘Highly Unlikely’ and ‘Unlikely’ were combined to report a negative response. Where respondents provided a free text response to a multiple-choice question, these were collated and either re-categorised into the original categories, or into new categories where appropriate. No missing data was amended or imputed, and results are reported as a proportion of the total responses to the individual question.

## Results

### Respondents and hospital characteristics

In the UK, 261 eligible participants were invited to take part in the survey. Eighty-seven declined to participate or did not respond; 174 participants were sent the questionnaire and 150 (86%) were returned. In Australia, 172 participants were contacted to complete the questionnaire, of these 10 declined and of the remaining 162, 120 (74%) returned the questionnaire. In total, 270 questionnaires were returned (overall response rate 80%).

Most respondents were nurses (77% in the UK and 85% in Australia) with a stroke-specific role within a stroke service (79% of UK and 73% of Australian respondents); most were female (86% in the UK and 92% in Australia); and about a third were between the ages of 41 and 50 years (28% UK and 30% Australian). Respondents mostly worked within an acute stroke unit. Table 1 shows key stroke service demographics.

**Table 1:** Key stroke service demographics

	UK n (%)	Australia n (%)
	150 (56)	120 (44)
**Hospital unit**		
Acute stroke unit	70 (47)	53 (44)
Ward with stroke beds	9 (6.0)	10 (8.3)
Integrated unit	48 (32)	17 (14)
Rehabilitation unit	21 (14)	35 (30)
Other	2 (1.3)	4 (3.3)
Not reported	0 (0.0)	1 (0.8)
**Hospital setting**		
Tertiary	60 (40)	55 (46)
Non-Tertiary with Emergency Department	67 (45)	47 (39)
Non-Tertiary without Emergency Department	20 (13)	12 (10)
Other	1 (0.7)	5 (4.2)
Not reported	2 (1.3)	1 (0.8)
**Stroke service availability^**		
Thrombolysis	124 (83)	84 (70)
Endovascular therapy	54 (36)	57 (48)
Neurovascular imaging	120 (80)	88 (73)
Telemedicine	81 (54)	65 (54)
Rehabilitation	130 (87)	78 (65)
Neurosurgery	55 (37)	56 (47)

^ More than one option permissible

Rates of collaborative completion of the survey were high in both countries (UK: 122, 81%; AUS: 93, 77%), with Speech and Language Therapists most often being called upon to support completion (UK: 40, 27%; AUS: 51, 43%) after senior nurses. This trend is likely due to the overarching aim of the survey, exploring oral healthcare practice. Table 2 shows the range of colleagues consulted in completion.

**Table 2:** Professions supporting survey completion

Roles supporting survey completion	UK n (%)	Australia n (%)
	150 (56)	120 (44)
Registered Nurse	97 (65)	65 (54)
Clinical Nurse Specialist	28 (19)	15 (13)
Clinical Nurse Consultant	4 (3)	14 (12)
Nurse Unit Manager	40 (27)	27 (23)
Nurse Practitioner (Stroke)	10 (7)	4 (3)
Clinical Nurse Educator	3 (2)	17 (14)
Stroke Liaison Nurse	7 (5)	6 (5)
Stroke Co-ordinator	10 (7)	10 (8)
Dentist	0 (0)	0 (0)
Oral/Maxillofacial Surgeon	0 (0)	0 (0)
Consultant: Neurologist/Geriatrician/Physician	14 (9)	5 (4)
Medical Registrar	2 (1)	2 (2)
Speech and Language Therapist/Speech Pathologist	40 (27)	51 (43)
Occupational Therapist	18 (12)	15 (13)
Physician Assistant/Associate	1 (1)	1 (1)
**Other**		
Dietitian	7 (5)	20 (17)
Healthcare Assistant	11 (7)	N/A
Mouth Care Specialist	3 (2)	N/A
Physiotherapist	2 (1)	1 (1)
**No response**	28 (19)	27 (23)

^ More than one option permissible

### Hydration care practice post-stroke

**Fig. 1:**
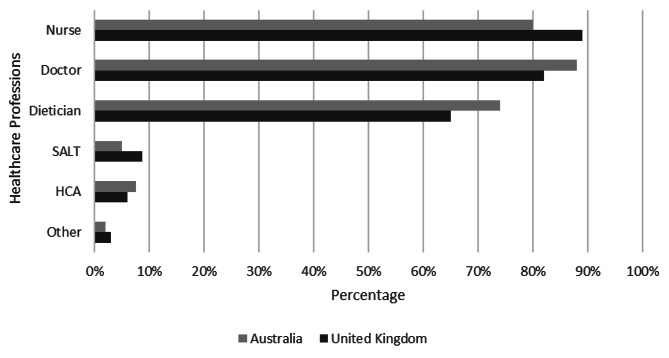
Healthcare professions most likely to assess hydration status

Almost all (258, 96%) of the surveyed hospitals stated that hydration status was assessed at some point during admission. In both countries, nurses (229, 85%), doctors (228, 84%) and dietitians (187, 69%) were most likely to assess hydration status (Fig. 1).

**Fig. 2:**
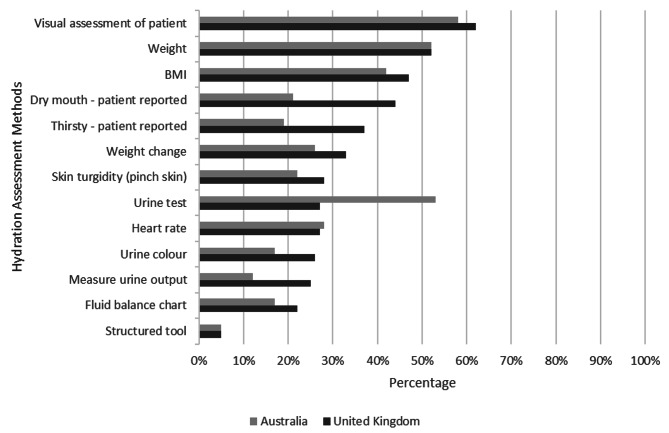
Most commonly used methods of hydration assessment on admission

The most common method of hydration assessment on admission was visual assessment of the patient (UK: 93, 62%; AUS: 70, 58%), followed by patient weight (UK: 78, 52%; AUS: 62, 52%) and BMI (UK: 70, 47%; AUS: 51, 42%). A range of other methods were used with similar frequency in both countries; however, urine tests were used more frequently in Australia (64, 53%) than in the UK (41, 27%) (Fig. 2). The most commonly utilised methods for daily assessment were: heart rate (UK: 109, 73%; AUS: 70, 58%); visual assessment of the patient (UK: 98, 65%; AUS: 73, 61%); patient reported dry mouth (UK: 97, 65%; AUS: 57, 48%); patient reported thirst (UK: 93, 62%; AUS: 52, 43%); and urine colour (UK: 84, 56%; AUS: 50, 42%). Weekly assessments were mainly focused on patient weight, BMI, and any changes in these. Additional assessments conducted in response to changes in patient status were urine tests, review of fluid balance charts, patient reported thirst, urine colour and output. Whilst blood tests were not routinely requested to assess hydration status at fixed time points, around half of all sites reported they would be requested as required.

In both countries, hydration management decisions were primarily made by physicians, who also took responsibility for the calculation of fluid intake requirements (UK: 126, 84%; AUS: 99, 83%). Over half (153, 58%) of all respondents stated daily monitoring of oral intake would take place during oral care assessments, though this was more usual in the UK (n = 97, 66%) than Australia (n = 56, 49%).

There was agreement regarding the preferred options for fluid replacement approaches with both the UK and Australia encouraging oral intake for patients without dysphagia (UK: 132, 88%; AUS: 111, 92%), and intravenous fluids being the first option for those with dysphagia (UK: 107, 71%; AUS: 82, 68%), followed by nasogastric (enteral) tube (UK: 93, 62%; AUS 73, 68%).

### Nutrition care practice post-stroke

Almost all (267, 99%) of the surveyed hospitals stated that nutritional status was assessed at some point during admission. Across all hospitals, nurses were most likely to complete nutrition assessments (245, 91%) followed by dietitians (209, 77%) and doctors (92, 34%). Other professions with a role in nutritional assessment included speech and language therapists (23, 9%), healthcare assistants (14, 5%), and others, for example mouthcare leads and student nurses (4, 2%).

Practice differed in the UK with a higher proportion of nurses (146, 97%) taking responsibility for the assessment of nutritional status compared to their Australian counterparts (99, 83%). Conversely, a greater proportion of dietitians (104, 87%) assessed nutritional status in Australia compared to the UK (105, 70%).

**Fig. 3:**
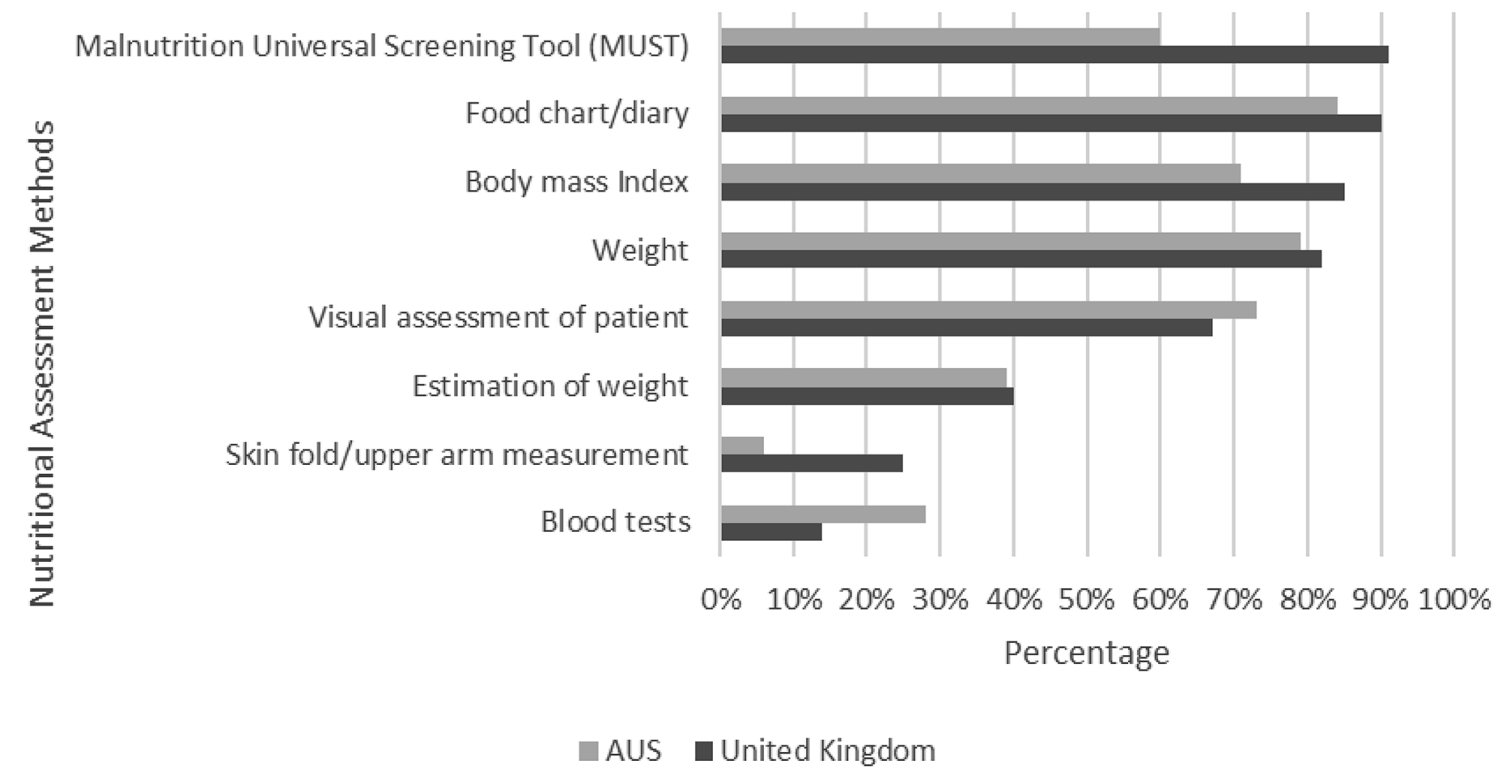
Measures used to assess nutritional status

In both countries the most commonly used methods for the assessment of nutritional status were: food chart/diary (UK: 135, 90%; AUS: 101, 84%), weight (UK: 123, 82%; AUS: 95, 79%), body mass index (BMI) (UK: 128, 85%; AUS: 85, 71%), and visual assessment (UK: 103, 67%; AUS: 87, 73%) (Fig. 3). The Malnutrition Universal Screening Tool (MUST) was more commonly used in the UK than in Australia (UK: 136, 91% vs. AUS: 72, 60%). In contrast, blood tests were more frequently used in Australia.

Nutritional status was mainly assessed on admission in both countries (220, 82%) with daily assessments only occurring in 31% (N = 83) of hospitals. Routine weekly assessment was more likely to occur in the UK than in Australia (UK: 116, 77% vs. AUS: 50, 42%). Assessment on referral occurred more frequently in Australia compared to the UK (AUS: 64, 53% vs. UK: 25, 17%). Similar patterns were observed across all clinical settings.

Where a patient was assessed as being over or underweight, the required calorie intake was determined through discussion with a dietitian (UK: 147, 98% vs. AUS: 116, 97%).

The routine documentation of hydration and nutrition care clinical assessments and care planning was similar for the UK and Australia and was most often captured in fluid balance charts (UK: 141, 94%; AUS: 110, 92%), food diaries/charts (UK: 142, 95%; AUS: 107, 89%), and patient medical notes (UK: 123, 82%; AUS: 108, 88%).

## Discussion

To our knowledge, this is the first concurrent survey conducted across the UK and Australia to identify and compare current hydration and nutrition care practices post-stroke in acute care and rehabilitation hospitals. The response rate across both countries was 80% (UK 86%; AUS 74%) in line with best practice to enhance rigour in survey research [32]. Dehydration and malnutrition are common, often unrecognised, problems in hospital patients with stroke, which are associated with increased mortality and poor outcomes [1, 33–35], and are important factors in the recovery of patients’ post-stroke.

In both Australia and the UK, hydration status was assessed on admission for the majority of patients. This follows Australian guideline recommendations that ‘hydration should be assessed, monitored and managed throughout hospital admission’ [20] and UK guidelines specifying that this should take place within 4 h of arrival [19]. UK guidelines go further, suggesting the use of ‘multiple methods’ for assessing hydration, yet no detail is provided to guide the optimum methods for use in practice. In our study, visual assessment, weight, and BMI were the most commonly used methods of hydration assessment on admission in both countries; urine tests were used more frequently in Australia, compared to the UK (53% vs 27%), and half of all hospitals reported that they would request blood tests to aid hydration assessment, often in response to a change in patient status. Although weekly monitoring of weight may be useful in the assessment of hydration status, admission measurements of weight, without a previous reference point, are unlikely to aid clinical decision making and diagnosis of dehydration. Similarly, the use of BMI as an admission assessment measure is also questionable given the lack of diagnostic utility in relation to hydration status. Whilst visual assessment of patients is of course valuable, a recent Cochrane systematic review of the signs and symptoms of impending and current water-loss dehydration in older patients found little evidence that any one sign, symptom, or test, including many that clinicians customarily rely on, has any diagnostic utility for dehydration [36]. While it is encouraging that our survey suggests admission hydration assessment practice is in line with guidelines, the methods utilised have been shown to be poor indicators of hydration status and further research is needed to determine the optimum methods of hydration assessment.

The assessment of hydration status was undertaken by nurses, doctors, and dietitians in both countries, and supported by various other professions including speech and language therapists and healthcare assistants. These findings reflect those in a previous study which found that the assessment, diagnosis, and management of dehydration requires complex multidisciplinary (MDT) teamwork [18]. Protocols to guide the optimum methods of hydration assessment may therefore benefit from a multi-disciplinary approach which utilises the skills and expertise of the team, maximising their individual contributions to improve care.

In this study, the most common method of documenting oral intake was the completion of fluid balance charts. However, in practice these are often incomplete and/or inaccurate, largely due to the inadequate training of, and poor communication between, nursing and healthcare staff [22, 37]. Practice in this area may therefore be improved by raising awareness regarding the importance of accurate recording, and the provision of training and protected time to improve completeness of documentation.

In-line with national clinical guidelines, the results of this study suggest that the nutritional status of patients was assessed on admission in the majority of hospitals. Despite structured screening tools being recommended for nutritional assessment post-stroke, the MUST was used more frequently in the UK than in Australia, potentially explained by the higher proportion of dietitians supporting nutritional assessment in Australia, and a range of assessment methods were more commonly used, such as food diaries, weight, BMI, visual assessment, and blood tests. As the Australian guidelines stipulate ‘staff should be trained in the use of a structured nutrition screening tool’, practice may benefit from efforts to implement this recommendation in routine care.

Decline in nutritional status post-stroke is multifactorial and affected by physical and functional impairments (dysphagia, upper limb weakness, postural control); cognitive and communication problems; changes in mood; and the side effects of medication; the impacts of which often vary throughout hospital admission. Therefore, nutritional status should be regularly assessed to ensure that nutritional intake is maximised to aid recovery and rehabilitation, and to prevent complications [38].

Nutrition guidelines recommend that patients should be assessed for the risk of malnutrition on admission and at least weekly thereafter [19, 20]. However, there was variability in practice with routine weekly assessment more likely to occur in the UK than in Australia (77% vs. 42%), and assessment on referral being more common in Australia (53% vs. 17%). Research has shown that the risk of malnutrition increases in the first ten days of admission [4], therefore patient outcomes have the potential to be improved if the rates of routine weekly nutrition assessments found in this study were increased.

The assessment of patients’ nutritional status was largely the responsibility of nurses (91%) and dietitians (77%). It was interesting to note that a larger proportion of dietitians assessed nutritional status in Australia compared to the UK and this may be due to local clinical protocols or the composition of stroke teams. In addition, the required calorie intake for patients assessed as being over or underweight were determined predominantly by dietitians. It is recognised that stroke services require dedicated dietetic support within a stroke specialist MDT to achieve best patient care [39] and there is evidence to suggest that the risk of malnutrition after stroke is minimised by dedicated dietetic input within the MDT, including the embedding of continued education and training, to ensure hydration and nutrition are everyone’s business [40]. Shortages, and inequitable distribution geographical distribution, of the dietetic workforce are reported in both the UK and Australia, presenting a barrier to safe and effective hydration and nutrition care post stroke [41, 42].

Considering the significant economic burden presented by post admission dehydration and malnutrition [1, 2], the development and implementation of evidence-based strategies to inform practice offers a cost-effective solution to improve patient outcomes and reduce healthcare expenditure.

### Strengths & limitations

The survey had a good response rate in both countries, which suggests that staff viewed hydration and nutrition care as an important topic and engaged with the study. However, the study used a self-reporting questionnaire which may have resulted in response (surveys posted to nurses rather than medical or allied health colleagues) and recall bias. Despite the respondents being encouraged to consult other team members, senior nurses were the profession most consulted which may have biased the results. Similarly, responses may represent only the respondents’ experiences and may not be fully reflective of wider MDT practices. The survey employed multiple-choice questions, with pre-defined answers informed by expert knowledge, literature, guidelines and research, which may have biased the results. However, respondents were able to provide alternative answers should they feel their practice was not reflected in the options available. Although many important topics were explored through the survey, the rationale underpinning hydration management and treatment decisions were not investigated. Due to the design and scope of the study, it was not possible to compare the findings with patient outcomes or experiences, which would be invaluable in future research to inform improvements in practice. Both Australia and the UK are classified as high-income countries with free, or low cost, access to healthcare, consequently the reults of this study are not necessarily generalisable to other settings, such as low and middle-income countries and those with private healthcare programmes. However, nutrition and hydration assessment and management are fundamental aspects of care across all settings, with relatively cost-effective solutions requiring few additional resources, and the findings may inform improvements for implementation in a variety of settings.

## Conclusion

This study demonstrates the key role of nurses in the assessment and management of post-stroke hydration and nutrition status, with doctors and dietitians supporting diagnosis and treatment care planning. We have found that there is variability in the timing and methods of assessment used in clinical practice, contrary to guideline recommendations. Hydration care could be improved through the development of a standardised approach to assessment, diagnosis, and treatment within the context of a multi-disciplinary stroke team, underpinned with the appropriate knowledge and skills. Whilst the assessment of nutrition is more often supported by a structured screening tool, practice could be improved by increased implementation of existing tools to standardise care. Further research is needed to identify the barriers and enablers to providing effective post-stroke hydration and nutrition care in hospital.

### Electronic supplementary material

Below is the link to the electronic supplementary material.

Supplementary Material 1

Supplementary Material 2

## Data Availability

The datasets used and/or analysed during the current study are available from the corresponding author on reasonable request.
