# Supplementary material for: Hydration and nutrition care practices in stroke: findings from the UK and Australia
Source: BMC Nurs. 2023 Oct 27;22:403. doi: 10.1186/s12912-023-01575-4 (PMC10604400; doi:10.1186/s12912-023-01575-4)
Supplement: Supplementary file 1 — Supplementary Material 1 [file 12912_2023_1575_MOESM1_ESM.pdf]

A				

Oral Health Care Practices in Acute Stroke Care

An international survey

A				

THANK YOU FOR TAKING PART IN THIS SURVEY

The questions in this survey refer to patients with stroke who are cared for in the stroke unit or in other wards.

We encourage that you **consult with your colleagues** who provide oral health care for acute stroke patients **to assist in the completion of the questionnaire**.

The survey results will provide an understanding of current oral care practices for stroke patients in hospital. This information will help to identify barriers and enablers to oral health care, inform whether specific education for oral health care is required and identify topics for future research

Some answers require more than one response. Please read the questions and instructions carefully and respond as accurately as possible.

Thank you again for your participation

A				

Section 1 Demographics and characteristics of person completing survey.

1. Please provide your job title. ***Please tick all that apply***

☐	Registered Nurse	☐	Dentist
☐	Clinical Nurse Specialist	☐	Oral/ Maxillofacial Surgeon
☐	Clinical Nurse Consultant	☐	Consultant: Neurologist/ Geriatrician/ Physician
☐	Nurse Unit Manager	☐	Medical Registrar
☐	Nurse Practitioner (stroke)	☐	Speech and Language Therapist/ Speech Pathologist
☐	Clinical Nurse Educator	☐	Occupational Therapist
☐	Stroke Liaison Nurse	☐	Physician Assistant/Associate
☐	Stroke Coordinator	☐	Other, ***please specify***:____________________________

2. Do you have a stroke-specific role within your facility or service?

If yes, tick one of the following:

☐	Stroke Coordinator	☐	Stroke Unit Director
☐	Stroke Liaison Nurse		
☐	Clinical Nurse Consultant		
☐	Other, ***please specify***: ____		

If no, tick box

☐ No

3. How long have you worked in your current role?

________ Years ________ Months

4. What is your gender?

☐ Male ☐ Female ☐ Other

5. What is your age?

☐ 21-30 years
☐ 31-40 years
☐ 41-50 years
☐ 51-60 years
☐ > 60 years

6. Please indicate below the roles of all those who may have helped you to complete the survey.

Please tick all that apply.

☐	Registered Nurse	☐	Dentist
☐	Clinical Nurse Specialist	☐	Oral/ Maxillofacial Surgeon
☐	Clinical Nurse Consultant	☐	Consultant: Neurologist/ Geriatrician/ Physician
☐	Nurse Unit Manager	☐	Medical Registrar
☐	Nurse Practitioner (stroke)	☐	Speech and Language Therapist/ Speech Pathologist
☐	Clinical Nurse Educator	☐	Occupational Therapist
☐	Stroke Liaison Nurse	☐	Physician Assistant/Associate
☐	Stroke Coordinator	☐	Other, ***please specify***:___________________________

7. What best describes the unit or ward where <u>you</u> work?

☐	Acute stroke Unit
☐	Ward with stroke beds, but not a ‘formal acute stroke unit’
☐	Integrated unit (acute and rehab beds in the same ward)
☐	Rehabilitation unit
☐	Other, ***please specify***:____

A				

Section 2 Hospital and stroke service characteristics.

8. What category below best describes your hospital setting?

☐ Tertiary referral, University or Teaching Hospital
☐ Non-tertiary, General, District or Community Hospital – with Emergency Department
☐ Non-tertiary, General, District or Community Hospital – without Emergency Department
☐ Other, ***please specify***:____

9. Please tell us about your stroke service:

(Please select one only)

☐ We have a dedicated stroke unit with clinicians who have stroke expertise
☐ We **do not** have a dedicated stroke unit, but ward(s) with stroke beds
☐ We are a free-standing rehabilitation hospital *(go to question 11)*

9a. Which of the options below are provided by your acute stroke service?
(Please select all that apply)

	Service available	*This is a 24/7 service* *Y*	*N*
Neurovascular imaging	○	○	○
Thrombolysis	○	○	○
Endovascular therapy	○	○	○
Neurosurgery	○	○	○
Telemedicine	○	○	○
Rehabilitation	○		

10. Please tell us about your rehabilitation service

(Please select one only)

☐ Rehabilitation ward within acute hospital in **same** building of **same** health campus
☐ Rehabilitation ward within acute hospital in **separate** buildings of **same** health campus
☐ Rehabilitation ward within acute hospital in **separate** buildings on a **separate** health campus
☐ Rehabilitation service within acute hospital (no dedicated beds, but ward(s) with stroke rehabilitation beds)

A				

Section 3 Oral health care practices for patients with stroke.

This section focuses on oral health care practices for patients with stroke in your stroke unit or ward. This section is **not** about swallow screening protocols.

11. Does your stroke unit or the ward where the majority of your patients with stroke are managed, have a protocol or guidelines about oral health care practices after acute stroke?

- ☐ Yes – stroke patient specific oral care protocol
- ☐ Yes – general oral care protocol for all patients
- ☐ No *(go to question 13)*
- ☐ Don't know *(go to question 13)*

12. How likely are clinical staff to use the oral care protocol?

- ☐ Highly Likely
- ☐ Likely
- ☐ Unsure
- ☐ Unlikely
- ☐ Highly Unlikely

13. Have staff working at your hospital received training in oral care provision in the last year?

- ☐ Yes
- ☐ No *(go to question 16)*
- ☐ Unsure *(go to question 16)*

14. Which staff groups working in stroke care have received training in oral care provision?

Please tick all that apply.

- ☐ Registered Nurse
- ☐ Enrolled Nurse
- ☐ Healthcare Assistant (UK)/ Assistant in Nursing (AUS)
- ☐ Physician Assistant/Associate
- ☐ Student Nurse
- ☐ Specialist Stroke Nurse (Clinical Nurse Consultant, Clinical Nurse Specialist, Nurse Practitioners)
- ☐ Speech and Language Therapist / Speech Pathologist
- ☐ Occupational Therapist
- ☐ Unsure
- ☐ Other, ***please specify***: ____

15. Who provided the training?

Please tick all that apply.

- ☐ Clinical Nurse Educator
- ☐ Nurse Practitioner
- ☐ Speech therapist/pathologist
- ☐ Specialist Stroke Nurse (Clinical Nurse Consultant, Clinical Nurse Specialist, Nurse Practitioner)
- ☐ Dentist/ Dental Hygienist
- ☐ Other hospital dental staff
- ☐ Nurse educator
- ☐ Informal (from other staff)
- ☐ External Health Professional/Educator
- ☐ Unsure
- ☐ Other, ***please specify***: ____

16. Are dental professionals employed by your hospital?

- ☐ Yes
- ☐ No
- ☐ Unsure

17. Do dental professionals support staff on your stroke unit or ward?

- ☐ Yes
- ☐ No *(go to question 21)*
- ☐ Unsure *(go to question 21)*

18. If yes, what best describes the service that dental professionals provide on your stroke unit or ward?

- ☐ Referral or on request
- ☐ Regular dedicated sessions
- ☐ Unsure
- ☐ Other***, please specify***:____

19. How often do you get help from dental professionals?

Please tick all that apply.

- ☐ Several times a week
- ☐ Once a week
- ☐ Once a month
- ☐ Once every few months
- ☐ Other:____________________________________

20. Which dental professionals provide this help?

Please tick all that apply.

- ☐ Patient's own dental practitioner
- ☐ Maxillofacial staff
- ☐ Dental hospital staff
- ☐ Dental hygienists
- ☐ Community dentists
- ☐ Don't know
- ☐ No dental professionals provided help
- ☐ Other, **please specify**:____________________________________

Section 4 Assessment of oral health care practices for stroke patients.

This section is about assessment and provision of oral care for stroke patients who are in your stroke unit or ward.

21. Does your ward or unit use an oral care assessment tool?

Please tick all that apply.

☐	No tool used
☐	The Holistic and Reliable Oral Assessment Tool (THROAT)
☐	Oral Health Assessment Tool (OHAT)
☐	Oral Assessment Tool (Eiler)
☐	Oral Cavity Assessment Tool (OCAT)
☐	Lockwood's Oral Health Assessment Tool (LOHAT)
☐	Beck Oral Assessment Scale (BOAS)
☐	Oral Assessment and Intervention tool (OAIT)
☐	Geriatric Oral Health Assessment Scale (GOHAI)
☐	Brief Oral Health Status Examination (BOHSE)
☐	Oral Assessment Guide (OAG)
☐	Rattenbury, Mooney, Bowen Mouth Assessment Tool (RMBMAT)
☐	Mouth Care Assessment Tool (MCAT)
☐	Daily oral health assessment (DOHA)
☐	Local area/hospital specific tool
☐	Unsure
☐	Other, ***please specify***:____

A				

22. How likely would an <u>oral care assessment</u> be undertaken at the following times?

Please tick one box on each line.

**Frequency of assessment**	**Highly Likely**	**Likely**	**Unsure**	**Unlikely**	**Highly Unlikely**	**N/A**
On admission to ward/unit						
Every nursing shift						
Daily						
Weekly						
As required or ad-hoc						
On discharge						
Other, ***please specify***:_____ _________________________ _________________________						

A				

23. How likely is each factor listed below to influence whether an oral care assessment is undertaken?

Please tick one box on each line.

**Patient factors**	**Highly Likely**	**Likely**	**Unsure**	**Unlikely**	**Highly Unlikely**
Dysphagia					
Aphasia					
Dysarthria					
Cognitive impairment					
Alert and able to self-manage					
Unconsciousness					
Physical impairment (upper limbs)					
Physical impairment (lower limbs)					
Nil by mouth					
Inattention/visual field problems					
Patient's poor motivation					
Malnourished					
Dehydrated					
Poor dental health					
Own teeth					
Dentures					
Older age					
Patient on medication that dries mouth					
Facial weakness					
Oxygen therapy					
Other, ***please specify*** ________________________________					
Other, ***please specify*** ________________________________					

24. How likely are the following professional groups to conduct an oral care assessment?

Please tick one box for each professional group.

**Professional group**	**Highly Likely**	**Likely**	**Unsure**	**Unlikely**	**Highly Unlikely**	**N/A**
Registered Nurse						
Enrolled Nurse/Advanced Diploma Nurse						
Assistant in Nursing/Health care assistant						
Student Nurse						
Clinical Nurse Consultant						
Specialist Stroke Nurse (including Clinical Nurse Consultant, Clinical Nurse Specialist, Nurse Practitioner)						
Nurse Practitioner						
Speech and Language Therapist/ Speech Pathologist						
Occupational Therapist						
Dieticians						
Doctor						
Dentist/ Dental Hygienist						
Oral/ Maxillofacial Surgeon						
Other, ***please specify*** ______________________________						
Other, ***please specify*** ______________________________						

A				

25. If a patient with stroke is incapable of independent oral care, how likely is each professional group to provide oral care?

Please tick one box for each professional group.

**Professional group**	**Highly Likely**	**Likely**	**Unsure**	**Unlikely**	**Highly Unlikely**	**N/A**
Registered Nurse						
Enrolled Nurse/Advanced Diploma Nurse						
Assistant in Nursing/Health care assistant						
Student Nurse						
Clinical Nurse Consultant						
Specialist Stroke Nurse (Clinical Nurse Consultant, Clinical Nurse Specialist, Nurse Practitioner)						
Nurse Practitioner						
Speech and Language Therapist/ Speech Pathologist						
Occupational Therapist						
Dieticians						
Doctor						
Dentist/ Dental Hygienist						
Oral/ Maxillofacial Surgeon						
Other***, please specify***: ______________________________						

26. If a patient is incapable of independent oral care, are family or carers (non-professionals) encouraged to <u>provide oral care</u> to patients with acute stroke?

☐ Yes

☐ No *(go to question 28)*

☐ Unsure *(go to question 28)*

A				

27. If yes, do they receive any oral care training or guidance from health professionals?

- [ ] Yes
- [ ] No
- [ ] Unsure

28. If a patient with stroke is incapable of independent oral care, how often are staff <u>expected</u> to perform the cleaning of natural teeth, dentures and oral soft tissue?

Please tick <u>one</u> option for each of the three columns.

**Option**	**Cleaning of natural teeth**	**Cleaning of dentures**	**Cleaning of soft tissue**
Three times a day			
Twice a day			
Once a day			
Weekly			
Never			
Unsure			
Other, **please specify**: ___________________________			

29. For patients incapable of independent oral care and who fall into any of the three groups in the table below, how often are staff <u>expected</u> to perform oral care for them?

Please tick one option for each of the three columns.

**Option**	**1. Nil by mouth**	**2. Modified diet**	**3. Normal diet**
Three times a day			
Twice a day			
Once a day			
Weekly			
Never			
Unsure			
Other, ***please specify***: ___________________________			

A				

30. If a patient can attend to their own oral care, how likely are they to be routinely assessed by staff on their ability to continue to perform their own oral care?

☐ Highly likely
☐ Likely
☐ Unsure
☐ Unlikely
☐ Highly unlikely

31. Are oral health care practices documented in each patient's medical records?

☐ Yes
☐ No *(go to question 35)*
☐ Unsure *(go to question 35)*

32. Where are oral health care practices documented and how likely are they to be documented?

Please tick one box for each document type.

**Document type**	**Highly Likely**	**Likely**	**Unsure**	**Unlikely**	**Highly Unlikely**
Dedicated oral care form					
Patient clinical/progress notes					
Care plan					
Clinical pathway					
Observation chart					
Other, ***please specify***: ________________________________					
Other, ***please specify***: ________________________________					

A				

33. How often are staff expected to document oral health care practices?

Please tick one box for each frequency of documentation option.

**Frequency of documentation**	**Never**	**Rarely**	**Sometimes**	**Often**	**Always**
On admission to ward/unit					
Every nursing shift					
Daily					
Weekly					
As required or ad-hoc					
On discharge					
Never					
Other, ***please specify***: ________________________________					

A				

34. What aspects of oral health care are documented and how likely are they to be documented?

Please tick one box for each oral care element.

**Oral care elements**	**Highly Likely**	**Likely**	**Unsure**	**Unlikely**	**Highly Unlikely**
Date/time oral care occurred					
Frequency of oral care (e.g. how often attended)					
Areas of the mouth cleaned (e.g. teeth, dentures etc.)					
Equipment used to perform oral care					
Who provided care (e.g. hospital staff; patient; carer)					
Whether an oral health care plan was developed					
All elements as above using a generic tick box option on a care plan or pathway that indicates 'all oral health assessment and care attended'					
Other, ***please specify***: ___________________________________					

Section 5 Provision of oral health care resources.

This section is about the equipment and resources available on your unit or ward to facilitate and support oral care for patients with stroke.

35. If patients do not have their own oral hygiene products, which of the following are provided on your stroke ward or unit?

Please tick all that apply.

- ☐ Manual toothbrush
- ☐ Electric toothbrush
- ☐ Toothpaste
- ☐ Foam swab
- ☐ Glycerine swab
- ☐ Bleach
- ☐ Mouthwash
- ☐ Dental floss
- ☐ Suction equipment
- ☐ Soft cloth/towel
- ☐ Tongue scraper,
- ☐ Suction toothbrush
- ☐ Denture brush
- ☐ Steradent
- ☐ Corsodyl/chlorhexidine
- ☐ Mouthwash tablets
- ☐ Sodium bicarbonate
- ☐ Saline/sodium chloride solution
- ☐ Sodium hypochlorite (Milton)
- ☐ Denture adhesive
- ☐ Ascorbic acid/Vitamin C
- ☐ Other decontaminants e.g. Antibiotic gel
- ☐ Other, ***please specify***:_________________

36. Which of the following are available for patients with a dry mouth?

Please tick all that apply.

- ☐ Artificial saliva
- ☐ Citric acid
- ☐ Lemon & glycerine swab
- ☐ Ascorbic acid/Vitamin C
- ☐ Pastilles/lollipops
- ☐ Oral fluid (water/tea/soft drink)
- ☐ Malic acid
- ☐ Chewing gum
- ☐ Nicotinamide/Vitamin B_3
- ☐ Pilocarpine
- ☐ Biotene
- ☐ None
- ☐ Other, ***please specify***:____

A				

Section 6 Factors influencing oral care provision.

This section focuses on your views about potential barriers to providing oral care to stroke patients.

37. Consider each statement below and indicate your level of agreement for each.

	**Statement**	**Strongly agree**	**Agree**	**Unsure**	**Disagree**	**Strongly disagree**
**Staff Factors**	Staff do not routinely document when oral care has been delivered to patients					
	I am satisfied with the level of oral care provided to patients in my ward/unit					
	Ward staff are too busy with other ward duties to conduct oral care					
	Staff shortages impact on staff capacity to deliver oral care					
	Nurses lack confidence in delivering oral health care					
	Oral care is perceived by nurses as less important than other aspects of patient care					
	Nurses lack awareness about the health benefits of oral health					
	There is a lack of evidence to support oral health care after stroke					
	I am happy with the level of oral health education provided on my ward/unit					
**Organisational factors**	There is variability and inconsistency in oral health care provision					
	No assessment tool is used on my unit/ward to guide oral care assessment					
	Carers/family members are encouraged to undertake oral health care					
	Oral care after stroke is a neglected area of practice					
	Safety issues are a concern for staff and patients when undertaking oral health care i.e. aspiration					
	There is a lack of hospital and/or ward protocols on oral health care for patients after stroke					
	There is a lack of equipment i.e. toothbrushes, mouth rinses, dental floss, suction, on my unit/ward					
	There is a lack of access to specialist dental care at my hospital					
	Pre-registration education and training of nurses in oral health care provision is inadequate					
	Post-registration education and training of nurses in oral health care is inadequate					
**Patient factors**	Difficulties communicating with stroke patients when attempting to deliver oral care is a barrier					
	Altered patient sensory perception is a barrier to oral care, i.e. hypersensitivity, pain, numbness					
	Stroke patients may have an altered sensation of thirst					
	It is difficult to provide oral care after stroke to patients with cognitive impairment					

A				

Section 7 Oral intake (hydration and nutrition).

This section focuses on practices relating to monitoring oral intake, nutritional and hydration status of stroke patients.

38. How likely are oral care assessments to include daily monitoring of oral intake?

- ☐ Highly likely
- ☐ Likely
- ☐ Unsure
- ☐ Unlikely
- ☐ Highly unlikely

39. Is nutritional status assessed at any time during the admission?

- ☐ Yes
- ☐ No *(go to question 43)*
- ☐ Unsure *(go to question 43)*

40. Who assesses nutritional status?

Please tick all that apply.

- ☐ Doctor
- ☐ Dietician
- ☐ Nurse
- ☐ Other, ***please specify***: ____
- ☐ Unsure

41. When and how often is nutritional status assessed?

Please tick all that apply.

- ☐ On admission
- ☐ Daily
- ☐ Weekly
- ☐ On referral
- ☐ Unsure
- ☐ Other, ***please specify***: ____

A				

42. Which of the following measure(s) are used to assess nutritional status?

Please tick all that apply.

- ☐ Visual assessment of patient
- ☐ Estimation of weight
- ☐ Scales
- ☐ Body mass index (BMI)
- ☐ Food chart/diary
- ☐ Other, ***please specify***:___________ ______________________________
- ☐ Blood tests:
  - ☐ Albumin
  - ☐ Pre albumin
  - ☐ Other:
- ☐ MUST (Malnutrition Universal Screening Tool)
- ☐ Skin fold thickness/upper arm measurement

43. If a patient is underweight or overweight, how is the required calorie intake for that patient determined?

Please tick all that apply.

- ☐ Ask dietician
- ☐ Calculate from weight *Please give calculation formula:*_____________________
- ☐ Calculate from BMI *Please give calculation formula:*_____________________
- ☐ Unsure
- ☐ Other, ***please specify***: ____

44. Is hydration status assessed?

- ☐ Yes
- ☐ No *(go to question 47)*
- ☐ Unsure *(go to question 47)*

45. By whom is hydration status assessed?

Please tick all that apply.

- ☐ Doctor
- ☐ Dietician
- ☐ Nurse
- ☐ Other, ***please specify***: ____
- ☐ Unsure

A				

46. **If hydration status is assessed, please indicate below which measures are used, and the frequency the assessments occur.**

Please tick all that apply.

**Hydration status measure**	**Admission**	**Daily**	**Weekly or more**	**Never**	**As required**	**Don't know**
Visual assessment of patient						
Skin turgidity (pinch skin)						
Thirsty - patient reported						
Dry mouth - patient reported						
Weight						
BMI						
Weight change						
Measure urine output						
Look at colour of urine						
Fluid balance chart						
Heart rate						
Structured tool: ***Please state name of tool*** ______________ ____________________________						
Urine tests: Specific Gravity						
Other: ***Please specify***_______ ___________________________						
Blood tests: Osmolality						
Osmolarity						
Urea: creatinine ratio						
Other: ***Please specify*** _______ ____________________________						
Bioelectrical impedance analysis (BIA) ***If BIA used, what is the definition of dehydrated in Hz Axillial moisture?***						
Other: ***Please specify***_______ ____________________________						

A				

47. If a patient is dehydrated, how is the required fluid intake amount determined?

Please tick all that apply.

- ☐ Ask physician
- ☐ Calculated from weight
- ☐ Calculated from BMI
- ☐ Calculated from blood tests
- ☐ Unknown
- ☐ Other, ***please specify***: ____

48. If fluid replacement is required, which approach would most often be used for each of the two patient populations shown in the table below?

Please tick one option for each column.

**Fluid replacement options**	**Patient without dysphagia**	**Patient with dysphagia**
Oral fluids		
Intravenous fluids		
Subcutaneous fluids		
Nasogastric/nasoenteric fluids		
PEG fluids		
Unsure		
Combination of approaches: ***please specify***______________________________ ____________________________________		

A				

49. How likely is monitoring of oral intake and/or hydration status to be documented in the following?

Please tick one box in each row.

**Source of oral intake/hydration status documentation**	**Highly Likely**	**Likely**	**Unsure**	**Unlikely**	**Highly Unlikely**
Dedicated oral care form					
Patient clinical/progress notes					
Care plan					
Clinical pathway					
Observation chart					
Fluid balance chart (intake and output)					
Food diary/chart					
Not routinely documented					
Other, ***please specify***:______________ __________________________________					
